# Relaxin-3/RXFP3 Signaling and Neuroendocrine Function – A Perspective on Extrinsic Hypothalamic Control

**DOI:** 10.3389/fendo.2013.00128

**Published:** 2013-09-18

**Authors:** Despina E. Ganella, Sherie Ma, Andrew L. Gundlach

**Affiliations:** ^1^The Florey Institute of Neuroscience and Mental Health, The University of Melbourne, Melbourne, VIC, Australia; ^2^Department of Biochemistry and Molecular Biology, The University of Melbourne, Melbourne, VIC, Australia; ^3^Florey Department of Neuroscience and Mental Health, The University of Melbourne, Melbourne, VIC, Australia; ^4^Department of Anatomy and Neuroscience, The University of Melbourne, Melbourne, VIC, Australia

**Keywords:** relaxin-3, oxytocin, arginine vasopressin, CRH, feeding, metabolism, stress, reproduction

## Abstract

Complex neural circuits within the hypothalamus that govern essential autonomic processes and associated behaviors signal using amino acid and monoamine transmitters and a variety of neuropeptide (hormone) modulators, often via G-protein coupled receptors (GPCRs) and associated cellular pathways. Relaxin-3 is a recently identified neuropeptide that is highly conserved throughout evolution. Neurons expressing relaxin-3 are located in the brainstem, but broadly innervate the entire limbic system including the hypothalamus. Extensive anatomical data in rodents and non-human primate, and recent regulatory and functional data, suggest relaxin-3 signaling via its cognate GPCR, RXFP3, has a broad range of effects on neuroendocrine function associated with stress responses, feeding and metabolism, motivation and reward, and possibly sexual behavior and reproduction. Therefore, this article aims to highlight the growing appreciation of the relaxin-3/RXFP3 system as an important “extrinsic” regulator of the neuroendocrine axis by reviewing its neuroanatomy and its putative roles in arousal-, stress-, and feeding-related behaviors and links to associated neural substrates and signaling networks. Current evidence identifies RXFP3 as a potential therapeutic target for treatment of neuroendocrine disorders and related behavioral dysfunction.

## Introduction

Precise regulation of complex neural circuits in the hypothalamus governs essential autonomic processes *and* associated behaviors, such as metabolism, growth, and feeding; stress responses, arousal, and locomotor activity; as well as reproduction and social/sexual behavior (1–7). These intrinsic and often interacting neural circuits utilize various neuroendocrine peptides/hormones, such as thyrotropin-releasing hormone (TRH), growth hormone-releasing hormone (GHRH), somatostatin, orexins, melanin-concentrating hormone (MCH), agouti-related peptide (AgRP), pro-opiomelanocortin (POMC) gene products [alpha-melanocyte-stimulating hormone (α-MSH)], neuropeptide Y (NPY), corticotropin-releasing hormone (CRH) and urocortins, gonadotropin-releasing hormone (GnRH), arginine vasopressin (AVP), and oxytocin (8–16). The majority of these peptides and hormones signal via G-protein coupled receptors (GPCRs) and often multiple receptors exist for different members of a peptide family or for the same peptide modulator [e.g., Ref. (17–19)].

This combination of a large number of ligands and multiple receptors results in a vast diversity in the potential regulation of different populations of hypothalamic neurons. For example, a recent survey revealed more than 300 different GPCRs are expressed by the heterogeneous neurons in the paraventricular (PVN) and supraoptic nuclei (SON) alone (20). This diversity of potential functional regulation provides a challenge for neuroscientists and neuroendocrinologists to document the anatomical distribution and dissect the primary and integrative actions of different signaling systems, both within hypothalamic circuits and via their descending and ascending inputs. Importantly, modern experimental approaches including conventional and viral-based tract-tracing (21) and other viral-based methods, such as optogenetics and DREADD technology (22–25), combined with molecular genetics and complementary methods for measuring changes in physiology and behavior, are successfully dissecting the role of individual neuron populations and the key mediators involved. In turn, this is allowing a reappraisal of the “dogma” related to the function of several established neural transmitter and hormone networks in the hypothalamus and the integration of new “chemical players” into the existing circuitry.

Just over a decade ago, the final member of the relaxin and insulin-like peptide superfamily was discovered and named H3 relaxin (human) or relaxin-3 (rodents), in line with the prior discovery and characterization of two other relaxin genes in humans (26). However, unlike its related peptide, H2 relaxin or relaxin, which is widely distributed within the brain *and* peripheral tissues [see Ref. (27, 28) for review], relaxin-3 was found to be most highly expressed in brain (26, 29). In 2003, GPCR135 (now known as RXFP3) was identified as the cognate relaxin-3 receptor (30, 31) and was shown to be highly localized in various rat brain areas (30, 32), which were later confirmed to contain relaxin-3-positive axonal projections and terminations (33). A similar central distribution of relaxin-3 neurons and projections to that reported in the rat was subsequently observed in the mouse [Ref. (34); Allen Brain Atlas^1^] and macaque brain (35, 36), suggesting that this neuropeptide system has been highly conserved throughout evolution. Indeed, bioinformatic studies revealed that a relaxin-3-like ancestral peptide gave rise to the relaxin and insulin-like peptide superfamily and its sequence has been highly conserved by strong purifying selection, consistent with a highly conserved function in the central nervous system (37, 38).

After their discovery, characterization of the neuroanatomical distribution of relaxin-3- and RXFP3-expressing neurons provided insights into putative functions of relaxin-3; and a growing number of experimental studies have subsequently confirmed roles for relaxin-3/RXFP3 signaling in arousal, feeding, stress responses, and cognition [see Ref. (39) for review]. Several of these actions of relaxin-3 likely involve effects on RXFP3-positive hypothalamic neuron populations. Therefore, in this article we will provide a summary of the hypothalamic distribution of RXFP3 mRNA and protein, and relaxin-3 projections, a concise review of experimental data indicating that this neuropeptide/receptor system is a modulator of hypothalamic function, and a perspective on the future studies required to better understand this system and to exploit its therapeutic potential.

## Neuroanatomy of the Relaxin-3/RXFP3 System

**Figure 1:**
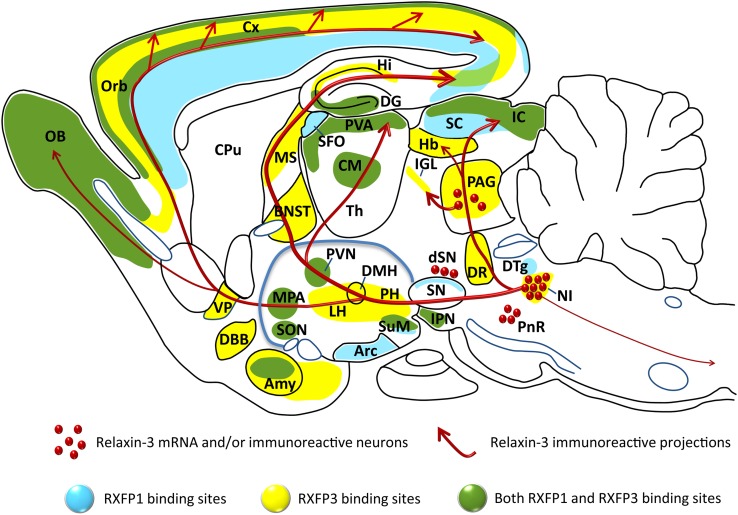
**Distribution of the relaxin-3 neural network and RXFP3 in relation to RXFP1, illustrated on a schematic parasagittal profile of the rodent brain**. Relaxin-3 immunoreactive fibers (red), RXFP3 binding sites (yellow), RXFP1 binding sites (blue), and regions that contain both RXFP1 and RXFP3 binding sites (green) are illustrated [see (32–34, 46, 62) and (28)]. Amy, amygdala; Arc, arcuate nucleus; BNST, bed nucleus of the stria terminalis; CM, centromedial thalamic nucleus; CPu, caudate putamen; Cx, cerebral cortex; DBB, diagonal band of Broca; DG, dentate gyrus; DMH, dorsomedial nucleus of hypothalamus; DR, dorsal raphe nucleus; DTg, dorsal tegmental nucleus; Hb, habenula; Hi, hippocampus; IC, inferior colliculus; IGL, intergeniculate leaflet; IPN, interpeduncular nucleus; LH, lateral hypothalamus; MPA, medial preoptic area; MS, medial septum; NI, nucleus incertus; OB, olfactory bulb; Orb, orbitofrontal cortex; PAG, periaqueductal gray; PH, posterior hypothalamus; PnR, pontine raphe; PVA, paraventricular thalamic area; PVN, paraventricular nucleus of hypothalamus; SC, superior colliculus; SFO, subfornical organ; SON, supraoptic nucleus; SN, substantia nigra; SuM, supramammillary nucleus; Th, thalamus; VP, ventral pallidum. Adapted from a figure kindly provided by Dr. Craig Smith (The Florey Institute of Neuroscience and Mental Health, Melbourne, Australia).

Relaxin-3 is a 5 kDa peptide that shares common structural features with all relaxin and insulin-like peptide family members – an A- and B-chain held together by three disulfide bonds (26, 40, 41). The native peptide is synthesized as a pre-prohormone that is subsequently cleaved by proteolytic processing of the signal and C-peptides to form the mature peptide [see e.g., Ref. (42, 43)]. Early *in situ* hybridization studies revealed that relaxin-3 mRNA was highly expressed by a cluster of neurons in the rat pontine central gray, identified as the *ventromedial dorsal tegmental area* (29), more commonly known as the *nucleus incertus* [NI; (44, 45)]. Smaller dispersed populations were also identified in the medial periaqueductal gray (PAG), pontine raphe (PR), and a region dorsal of the substantia nigra (dSN) in the rat (33, 46), mouse (34), and macaque (36) (Figure 1). Ultrastructural analysis of relaxin-3 immunoreactivity in the rat NI identified the peptide in the rough endoplasmic reticulum and Golgi apparatus in the cell soma and within dense-core vesicles adjacent to synapses in nerve terminals of distant target regions such as the lateral hypothalamus (46) and medial septum (47), indicating that relaxin-3 is processed and released as a transmitter.

The efferent and afferent connections of the rat NI have been characterized (44, 45, 48–50) and many NI projection target regions, including the hypothalamus, contain relaxin-3 immunoreactive fibers and terminals, and neurons expressing RXFP3 (33, 50) (Figure 1; Table 1), suggesting many of these areas are innervated by NI relaxin-3 neurons. Not surprisingly, NI relaxin-3 neurons have been the focus of the majority of functional studies to date, which indicate they are highly responsive to neurogenic stressors and CRH (46, 51–53).

**Table 1:** **Comparative distribution of relaxin-3 and its receptor, RXFP3, in hypothalamic regions of rat and mouse brain**.

Hypothalamic area/nucleus	RLN3-LI		RXFP3 mRNA		RXFP3 binding sites	
	Mouse	Rat	Mouse	Rat	Mouse	Rat
Anterior hypothalamic n.	−/+	+ +	+	+ +	−/+	+
Arcuate n.	−	+	+	+	−	−/+
Dorsomedial hypothalamic n.	+	+ ++	+	+ ++	+	n.r.
Lateral hypothalamic area	+ ++	+ ++	+ +	+ +	+	+
Lateral mammillary n.	+ +	+	−/+	−/+	−	−
Lateroanterior hypothalamic n.	−/+	+ +	+	+ +	−	n.r.
Medial mammillary n.	+	+ +	−	+	−	−
Paraventricular hypothalamic n.	+	+ +	+ ++	+ ++	+ ++	+ ++
Periventricular hypothalamic n.	−/+	+	+ ++	+ +	+ +	+ +
Posterior hypothalamic area	+ ++	+ ++	+ +	+	+	n.r.
Premammillary n.	+	+ +	+	+	+	n.r.
Preoptic area, lateral	+ +	+ ++	+ +	+ +	+ +	+
Preoptic area, medial	−/+	+ +	+	+ ++	−/+	−/+
Suprachiasmatic n.	+	−/+	+	−/+	−	−
Supramammillary n.	+ ++	+ ++	+ +	+ +	−/+	−
Supraoptic n.	+ ++	+ +	+ ++	+ ++	+ +	+ ++
Tuberomammillary n.	+	+ ++	+	+ ++	+	n.r.
Ventromedial hypothalamic n.	+/+ +	+ +	+	+ +	+	n.r.

Relative abundance values are given: − not detectable, + low density, + + moderate density, + ++ high density, n.r. not reported). Adapted from Ma et al. (33), Smith et al. (34), Sutton et al. (32), Tanaka et al. (46), Allen Brain Institute Brain Atlas (http://mouse.brain-map.org/experiment/show/71358555). RLN3-LI, relaxin-3 like immunoreactivity.

Less is known about the connections, regulation and function of other relaxin-3 neuron populations, but a recent study demonstrated that PAG relaxin-3 neurons strongly innervate and modulate neuronal activity in the intergeniculate leaflet [IGL; (46, 54, 55)], a region that is known to contribute to the regulation of arousal and circadian activity (56–58). In brain slice studies, patch-clamp recordings of IGL neurons, revealed that activation of RXFP3 by bath application of the agonist peptide, R3/I5, produced depolarization of identified NPY-containing neurons (55), which are known to project to the suprachiasmatic nucleus via the geniculohypothalamic tract (56, 58). Neurons in the IGL also project to a number of other hypothalamic areas, including the anterior and lateral hypothalamic areas, and the dorsomedial nucleus (58).

There are, however, several brain regions like the aforementioned IGL and the amygdala that contain dense relaxin-3 immunoreactivity and/or RXFP3, but sparse NI projections; suggesting they are also more strongly innervated by other relaxin-3 populations. In fact, there is anatomical evidence, chiefly from neural tract-tracing studies, to suggest the various RXFP3-positive regions in the hypothalamus are also innervated by relaxin-3 neurons in the NI *and* other relaxin-3 groups. For example, a recent study of brainstem inputs to the PVN and surrounding area in the rat (59) revealed projections from the medial PAG, PR, and “dorsal to substantia nigra” regions, which contain relaxin-3 neurons.

RXFP3 has been localized in various subregions of the hypothalamus in the rat (32, 33) mouse (34, 60) (Table 1), and macaque (36). The highest densities are present in the PVN, SON and adjacent medial (MPO) and lateral preoptic (LPO) nuclei, but there are RXFP3-positive neurons in other hypothalamic areas, including the periventricular nucleus (33, 34) (Table 1), which is consistent with a putative role of relaxin-3/RXFP3 signaling in the control of a range of homeostatic and autonomic behaviors via modulation of related hypothalamic networks.

## Relaxin-3 Receptor Binding and Activation in Brain

Effects of endogenous relaxin-3 are predicted to be mediated by its cognate receptor, RXFP3, but relaxin-3 is also an agonist at the relaxin-family receptors, RXFP1 and RXFP4, when administered at pharmacological doses, albeit with lower potency than at RXFP3 (30, 61). RXFP1 and RXFP4 are likely expressed in the human brain, along with RXFP3, but based on animal studies these receptors and the peptides which bind and activate them (relaxin, insulin-like peptide 5, and relaxin-3, respectively) are expressed in quite distinct regions of the brain and at very different levels [e.g., Ref. (26, 29, 30, 33, 46, 62)], so a current working hypothesis that can be tested is that these receptors mediate distinct functional effects in the brain, which modulate different homeostatic processes and behaviors.

Cell signaling events associated with the relaxin-family receptors have been studied in different cell lines transfected with the human receptors (28, 63) and activation of RXFP1 and RXFP3/4 by H3 relaxin leads to different intracellular responses *in vitro*. In Chinese hamster ovary (CHO) cells, RXFP3 and RXFP4 couple to the inhibitory G_αi_/G_αo_-protein system and receptor activation leads to sequestration of these G-proteins and inhibition of adenylate cyclase (AC), and subsequent cAMP accumulation (30, 64). The intracellular signaling pathway of the relaxin-3-RXFP3 interaction has also been studied in the SN56 neuronal-like cell line, in which the G_αi_/G_αo_ pathway was recruited, suggesting an inhibitory intracellular pathway may be activated *in vivo* when relaxin-3 binds to RXFP3-expressing neurons within the brain (63), an idea that can be tested in different functional networks [see Ref. (54, 55)]. In contrast, RXFP1 activation by either relaxin or relaxin-3 in mammalian cell expression systems initiates a downstream accumulation of cAMP, as it is predominantly coupled to the stimulatory G_s_-protein. These *in vitro* studies suggest activation of different receptors may lead to different downstream effects in neurons *in vivo*. Little is known, however, about the native intracellular signaling of any of the relaxin family of receptors (RXFP1–RXFP4) in specific neuronal populations, apart from a recent *in vitro* study that revealed activation of RXFP3 by the agonist peptide, R3/I5 (65), produced depolarization of identified NPY-containing neurons and hyperpolarization of adjacent non-NPY neurons (55).

Although there is no definitive evidence that major biological effects mediated by relaxin-3 are caused by activation of either RXFP1 and/or RXFP4, the ability of relaxin-3 to activate RXFP1 and RXFP4 as well as RXFP3 must be considered as a confounding factor when using pharmacological doses of peptides *in vivo*, in attempts to study neuropeptide function in the rodent. From a practical viewpoint, the rat is suited to studies of the neurobiology of relaxin-3/RXFP3 signaling, since RXFP4 is a pseudogene in this species, and so not a “confound.” However, *in situ* hybridization and radioligand binding site studies indicate RXFP1 is expressed in the rat brain in a number of regions positive for RXFP3, including the cerebral cortex, amygdala, thalamus, and hypothalamus (32, 33, 62). Consequently RXFP1 activity must be considered in studies of exogenous relaxin-3 peptide administration [e.g., Ref. (40, 66–70)]. The relative degree to which RXFP1 activation has impacted outcomes in studies of relaxin-3 actions within the hypothalamus and other brain areas is hard to gage, although in some cases comparative effects of relaxin were reported [see Ref. (71) below]. Fortunately, the development more recently of agonist and antagonist peptides which selectively activate or inhibit RXFP3 [e.g., Ref. (65, 72–74)] has facilitated investigations of the behavioral and physiological effects of specific relaxin-3/RXFP3 interactions. Indeed, several studies have used these peptides to assess relaxin-3/RXFP3 related functions associated with the hypothalamus, although their use is not as widespread as it might be [e.g., Ref. (69, 70)], particularly in light of the availability of a chemically less-complex, single-chain peptide antagonist for RXFP3 (73).

## Actions of the Relaxin-3/RXFP3 System – Focus on Hypothalamus

### Feeding and energy balance

The PVN and arcuate nucleus (ARC) are two hypothalamic nuclei which tightly regulate food intake and energy homeostasis [e.g., Ref. (3, 4, 12, 15, 75)]. Relaxin-3 immunoreactivity and RXFP3 mRNA and binding sites have been identified in the PVN and ARC (30, 32, 33) (Table 1) and extensive research has demonstrated that H3 relaxin can alter feeding and appetite in rats [see Ref. (76)]. Central (icv) administration of H3 relaxin caused hyperphagia in satiated male Wistar rats in the first hour after treatment in both the light and the dark phase, while equivalent doses of H2 relaxin did not produce a similar effect (66). The doses of H3 relaxin administered icv (180 pmol) or directly into the PVN (18 pmol) at which a significant increase in feeding was observed (see below) were similar to doses of other “feeding” peptides which elicit an increased feeding response upon similar administration [e.g., NPY ∼80 pmol iPVN; (77)], consistent with a physiological role for relaxin-3/RXFP3 signaling in modulating appetite and feeding behavior. Importantly, icv administration of the selective agonist, R3/I5, produced an increase in first-hour food intake and this effect was inhibited by co-administration of the selective antagonist, ΔR3/I5 (72), further suggesting RXFP3 involvement. In these feeding studies, the food intake was monitored for only 24 h post-injection, as it was predicted the injected peptide would be quite rapidly degraded by proteolysis. In a study of longer term effects of relaxin-3 on feeding and body weight, H3 relaxin (600 pmol/day) was infused icv into rats for 14 days via osmotic mini-pump, which produced a significant increase in food intake and body weight gain, and plasma leptin and insulin levels, compared to vehicle (78). This data indicated that the relaxin-3 induced increase in food intake could be sustained and lead to an increase in body weight and associated biochemical changes in the rat.

Given the primary role of the hypothalamus in energy balance, the effect on feeding of local hypothalamic injections of relaxin-3 was assessed. Acute H3 relaxin injection into the PVN (iPVN) increased food intake over the first hour (67). Sub-chronic iPVN H3 relaxin administration in *ad libitum* fed rats also produced an increase in food intake and cumulative body weight gain *c.f.* vehicle (67). These authors also used “Fos-activation” mapping after icv administration of H3 relaxin to identify activated hypothalamic nuclei. In addition to the PVN, the SON, ARC, and anterior preoptic area displayed increased Fos staining (79). When injected directly into these and other hypothalamic nuclei H3 relaxin stimulated a significant increase in food intake within the first hour, relative to control (79), suggesting multiple hypothalamic nuclei may mediate these potent orexigenic effects. Unfortunately the potential involvement of RXFP1 activation in the observed effects cannot be reliably excluded, as RXFP3-selective peptides were not utilized. It is also possible the injected peptide is able to diffuse from the injection site to adjacent areas that are primarily involved in the activation of feeding, although some targeted sites were not associated with feeding (79).

In order to circumvent issues associated with acute peptide administration and cross-reactivity of H3 relaxin at RXFP1, we used a viral strategy to investigate the effect of chronic R3/I5 mediated RXFP3 activation within the hypothalamic PVN (43). Using a recombinant adeno-associated virus (rAAV) engineered to locally secrete bioactive R3/I5 (rAAV-R3/I5), we demonstrated an increase in food intake in the R3/I5 expressing rats (∼5.2 g/day more than control) which was sustained for up to 2 months, leading to an ∼23% increase in cumulative body weight gain (43). In an attempt to identify targets of RXFP3 activation within the hypothalamus, levels of mRNA for a number of genes were also assessed in dissected hypothalamic tissue blocks using quantitative reverse transcription PCR. Notably, no major differences were identified in expression of some “major” feeding peptide genes (NPY, AgRP, POMC) between rAAV-R3/I5 and rAAV-control treated groups, whereas the levels of oxytocin and AVP mRNA were altered. This is, however, consistent with the strong expression of RXFP3 by neurons in the PVN and SON (30, 32, 33) (Table 1), which contain oxytocin- and AVP-containing magnocellular neurons. Chronic viral-mediated expression of R3/I5 in the hypothalamus for up to ∼14 weeks led to a marked reduction in oxytocin mRNA expression in PVN (∼50%) and a smaller reduction in AVP (∼25%), compared to control expression (43). These data suggest the acute orexigenic effect of RXFP3 activation may be mediated by changes in oxytocin release (and perhaps AVP) [see Ref. (80)], an idea that can be tested experimentally. Furthermore, there is a substantial literature relating to oxytocin as a satiety factor that also supports this putative acute and chronic mechanism of action.

### Interaction with oxytocin and arginine vasopressin systems in the PVN

Oxytocin is a peptide hormone highly expressed in magnocellular and some parvocellular neurons of the hypothalamic PVN and SON (81–83). Oxytocin is classically known as a reproductive hormone with roles in parturition, lactation, sexual behavior, and pair bonding and attachment [see Ref. (84, 85) for review]. However, early studies revealed that central administration of oxytocin dose-dependently reduced food consumption and time spent eating and increased the latency to the first meal in pre-fasted rats; an effect prevented by co-administration of an oxytocin receptor antagonist (86). A number of studies have since confirmed central oxytocin administration inhibits food intake, strengthening the hypothesis that oxytocin serves a key role in appetite control (87–89). More recent data is consistent with this view, as oxytocin null mutation mice display enhanced intake of sweet and non-sweet carbohydrate solutions (90, 91) and develop late-onset obesity (92).

The magnitude of the increase in feeding and body weight gain observed in our hypothalamic R3/I5 expression studies (43) was modest relative to those seen after similar viral-mediated NPY and AgRP over-expression, consistent with actions independent of direct effects on these neurons. The down-regulation of oxytocin and AVP mRNA expression observed (43), suggests the R3/I5 agonist peptide is activating RXFP3 on oxytocin- and AVP-containing neurons, which results in downstream effects on the transcription of oxytocin and AVP mRNA in these neurons (e.g., Ref. (93, 94)] and subsequent effects on production and release of this anorexigenic hormone. If RXFP3 associated cell signaling in these neurons is similar to effects reported *in vitro*, this could be associated with reduction in cellular cAMP levels (via inhibitory G_i_-protein coupling) and inhibition or hyperpolarization of target neurons – a possibility that can be assessed experimentally using *in vitro* or *in vivo* electrophysiological and biochemical methods [e.g., Ref. (55, 95)].

It is also necessary to establish whether it is the proposed reduction in oxytocin production and release which produces the observed increase in food intake in AAV-R3/I5-treated rats, or whether the chronic increase in food intake caused by hypothalamic RXFP3 activation over time leads to down-regulation of oxytocin mRNA via a distinct mechanism. This could be explored by conducting acute peptide administration studies or shorter time course viral infusion studies, and assessing oxytocin mRNA, peptide and release levels, before any marked changes in body weight have occurred.

Indeed, a recent study examined the effect of icv H3 relaxin on anxiety-like behavior in rats and observed an anxiolytic effect in the elevated plus maze test and the shock probe-burying test (70), consistent with our studies demonstrating that icv administration of a selective RXFP3 agonist peptide reduced anxiety-like behavior in the light-dark box and elevated plus maze (96). Notably, these authors used microarray and peptidomics approaches to identify associated downstream signaling targets in the hypothalamus altered by icv H3 relaxin and detected a relatively acute (6–24 h) and quite specific *increase* in the level of hypothalamic oxytocin mRNA and peptide levels (70). This data is, however, more consistent with the ability of H3 relaxin to *activate* neurons via RXFP1 [see e.g., Ref. (40, 67, 68)] rather than via RXFP3, which based on predicted signaling (28, 63) might be expected to decrease oxytocin neuron activity (and mRNA and peptide levels), as seen in our study (43). Therefore, further studies are required to identify specific effects of RXFP3 (and RXFP1) activation in hypothalamus and other areas in altered feeding and metabolism and anxiety-like behavior (76, 96).

Further evidence for an association of a down-regulation/inhibition of PVN oxytocin activity with a hyperphagic phenotype was reported recently. Optogenetic electrophysiological studies revealed that GABA/AgRP neurons in the ARC project to a population of oxytocin neurons in the PVN and strongly inhibit their activity, and this suppression of oxytocin neurons by AgRP neuronal activation drives evoked feeding (23). This study also demonstrated that increased food seeking and consumption in response to GABA/AgRP neuron activation is similarly induced by suppressing the activity of the PVN neurons that (selectively) express the *single-minded 1* (SIM1) transcription factor (23, 97).

Importantly from a clinical perspective, a number of disease states in which hyperphagia is a symptom are associated with hypothalamic oxytocin dysregulation. A small population of PVN oxytocin neurons is selectively lost in Prader–Willi syndrome, which is a condition involving insatiable hunger (98); and disruption of synaptic release of hypothalamic oxytocin results in overeating (99). Oxytocin deficits in SIM1 haploinsufficient mice and mutations in the SIM1 gene in humans lead to hyperphagic obesity (97, 100, 101). In the mouse model, an ∼80% reduction in oxytocin and ∼30% reduction in AVP was observed (97). These studies illustrate that modulation of relaxin-3 signaling and associated alterations in oxytocin neuron activity may be a fruitful area to explore for treating disease states associated with eating disorders. For example, a recent clinical cross-sectional study reported that female patients with anorexia nervosa, characterized by food restriction, low weight, and hypoleptinemia, had higher mean circulating oxytocin levels than healthy controls at all times assessed (102). The severity of disease psychopathology was also positively associated with circulating oxytocin levels (102).

### Hypothalamic-pituitary-adrenal axis and stress responses

Integration of the stress response via the hypothalamic-pituitary-adrenal (HPA) axis occurs via interaction between brain areas which are sensitive to stress and neuroendocrine neurons of the hypothalamic PVN, particularly those in the parvocellular region producing CRH [see Ref. (1, 6, 103) for review]. CRH stimulates the secretion and synthesis of adrenocorticotropin hormone (ACTH) from the pituitary and is the main regulator of HPA axis activity during stress.

Early regulatory studies revealed that neurons in the NI and specifically relaxin-3 expressing neurons respond to stress and CRH (46, 104), and that relaxin-3-containing neurons in the NI express CRH type 1 receptors (CRH-R1) (46). Upon icv administration of CRH, 65% of relaxin-3-positive neurons underwent activation (detected using Fos-immunostaining 2 h post-infusion) (46). Electrophysiological characterization of NI neurons revealed that a significant population increased firing following icv administration of CRH, of which the majority were relaxin-3-positive, though an almost equal number of non-relaxin-3 neurons exhibited a decrease in firing in the anesthetized rat (53). These findings are consistent with semi-quantitative immunohistochemical studies of the NI revealing this heterogeneous neuron population consists of relaxin-3 neurons that all co-express CRH-R1, though not all CRH-R1 contain relaxin-3, in addition to a significant population that are CRH-R1 negative (53). Rats tested in a water immersion-restraint stress paradigm, displayed increased Fos-immunostaining and an up-regulation of relaxin-3 mRNA in NI neurons (46). Subsequently, the effect of a repeat forced swim on relaxin-3 expression in the NI was examined and led to a rapid increase in relaxin-3 mRNA expression (51). This increase was largely mediated by CRH activation of CRH-R1 located on NI neurons, as pre-treatment with the CRH antagonist, antalarmin, reduced the increase in relaxin-3 mRNA expression by 70–80%. Levels of relaxin-3 heteronuclear (hn) RNA were also increased in NI neurons after the repeat forced swim (51). Changes in hnRNA are a measure of transcriptional activity and are thought to reflect the level of encoded peptide synthesis (105), suggesting in this case, an increase in relaxin-3 utilization.

Initial insights into the hypothalamic action of relaxin-3 in relation to the stress response have been obtained by monitoring the effect of icv administration of relaxin-3 in male rats (69). Increased Fos staining in the PVN and SON was observed 1 h post-administration and levels of c-*fos* and CRH mRNA in the PVN were also increased 2 h after H3 relaxin administration. Central H3 relaxin administration also elicited an increase in circulating plasma ACTH (69). These data *suggest* a role for relaxin-3 in the acute hypothalamus-pituitary CRH-ACTH system response, but these studies did not directly identify the presence of RXFP3 on CRH neurons or measure the direct acute excitatory or inhibitory effect of RXFP3 activation on these neurons [see Ref. (55)].

Notably, a recent report also suggests sex-specific regulation of CRH and relaxin-3 systems in response to combined stressors. Chronically stressed and repeatedly food-restricted female rats consumed more standard chow during recovery and had an increased bodyweight relative to controls, whereas male rats exposed to this regime had a reduction in bodyweight (106). Stressed/food-restricted female rats had elevated plasma corticosterone and low PVN CRH mRNA levels. CRH neurons in the medial preoptic area were identified as a source of increased CRH production/release during stress in female brain, i.e., CRH mRNA levels in this area were – higher in female than male rats, increased by chronic stress, and increased in female, not male, rats after repeated food restriction (106).

Further studies are now required to determine precisely how the robust, consistently observed stress and CRH-induced activation of NI and relaxin-3 neurons (46, 51, 53, 106) directly or indirectly activates (or possibly inhibits) the PVN and the main components of the HPA axis; and whether and how these processes are regulated by steroid and other hormones under different conditions.

### Effects on reproductive neuroendocrine systems

Preliminary studies have indicated a putative role for relaxin-3 in reproductive physiology. Following injection into the PVN and surrounds, H3 relaxin increased levels of marker hormones of the hypothalamic-pituitary-gonadal (HPG) axis (71, 107); and anatomical studies have identified RXFP3 in many areas in the hypothalamus relevant to reproductive neuroendocrinology, including the preoptic area and the PVN and SON (33). H3 relaxin administered icv or iPVN significantly increased plasma luteinizing hormone (LH) levels 30 min post-injection in male Wistar rats, an effect blocked by peripheral pre-treatment with a GnRH antagonist, consistent with increased central GnRH release (71). In these initial studies, H2 relaxin administration via the same routes produced a small, non-significant increase in LH, suggesting a stronger relaxin-3/RXFP3-mediated effect (71). Activity of the endogenous peptide at RXFP1 cannot be completely excluded from an involvement in these effects, however, as RXFP1 is expressed in the anterior hypothalamus and PVN of the rat (62). If so, high levels of endogenous relaxin-3 may act via RXFP1 to stimulate the HPG axis, an idea that could be tested experimentally. It would also be of interest to observe the effects of icv or iPVN administration of RXFP3-selective peptides (65, 72–74) on the HPG axis in female rats to check for similar hormone changes to those observed in male rats.

Notably, treatment of mouse hypothalamic neuron-like (GT1–7) cells or hypothalamic explants with synthetic H3 relaxin, produced a dose-dependent increase in secretion of GnRH (71). However, it is again unclear if this is an RXFP3-mediated effect, as GT1–7 cells and hypothalamic explants also express RXFP1; and H3 relaxin binds and activates these receptors. As these cells express both RXFP1 and RXFP3, the relaxin-3 mediated secretion of GnRH should be assessed for sensitivity to blockade with an RXFP3 antagonist (71–73).

In our recent study, we assessed the effect of chronic (∼14 weeks) viral-mediated expression of R3/I5 in the hypothalamus on GnRH mRNA levels (43). Although the difference between groups was not statistically significant, there was a trend for increased GnRH mRNA levels in AAV-R3/I5-treated compared to control rats, with considerable variability in individual values. Given the dispersed nature of GnRH expressing neurons in the hypothalamus, the variability observed suggests that different numbers (populations) of GnRH expressing neurons may have been dissected in these assays and/or there may be a genuine increase in GnRH transcription as a result of the treatment. This important question needs to be further investigated using suitable quantitative methods, in conjunction with further assessments of relaxin3/RXFP3 indices relative to GnRH and other reproductive peptides and receptors during different stages of pregnancy, birth and lactation.

### Other hypothalamic sites of relaxin-3/RXFP3 action

The relaxin-3/RXFP3 system may have actions in other hypothalamic nuclei, demonstrated by the presence of both relaxin-3 immunoreactive fibers and RXFP3 mRNA/protein in the lateral and medial preoptic areas, anterior, posterior, dorsomedial, and ventromedial regions, and the adjacent supramammillary nucleus (SuM) (33) (Table 1). The functions of these regions will be briefly reviewed, in view of the postulated role of relaxin-3 in arousal, feeding, behavioral state, and cognition (39).

The SuM receives a moderate relaxin-3 innervation in the rat (33) and the mouse (34), and this nucleus represents a major target of the peptide network in the macaque brain (35). In contrast to other “hypothalamic” regions, this nucleus is best characterized as a key input to and modulator of the hippocampus and hippocampal theta rhythm (108, 109), a synchronous activity between 4 and 12 Hz associated with active waking behavior and REM sleep (110), mnemonic processing (111, 112), spatial navigation and exploration (113), and sensorimotor integration (114, 115). There is growing evidence that the NI is a key player in mediating brainstem-elicited theta rhythm (47, 53, 116). In addition to projections to the SuM, there are strong relaxin-3-containing NI projections to other regions subserving theta rhythm generation, such as medial septum (48–50), *reticularis pontine oralis*, and the hippocampus, as well as the median raphe, which is involved in theta desynchronization [see (52) for review]. In fact, relaxin-3 projections to the medial septum have been shown to promote hippocampal theta rhythm and are necessary for normal spatial navigation of rats in a spontaneous alternation task (47). Thus, the SuM is likely a further node at which this neuropeptide system acts to regulate hippocampal theta activity and associated cognitive/autonomic processes.

The lateral and medial preoptic areas of hypothalamus are also rich in relaxin-3 projections and RXFP3. The LPO area contains populations of neurons that have identified roles in thermoregulation (117, 118) and sleep-wakefulness (ventrolateral neurons) (119), so it will be of interest, to assess the precise topography of RXFP3 in these areas and to assess the effects of RXFP3 activation/inhibition in these regions on these physiological parameters, particularly as our laboratory has anatomical and functional data consistent with effects of relaxin-3 on sleep-wake activity in mice (34, 120). Similarly, as discussed, the role of relaxin-3/RXFP3 signaling in the medial preoptic area may be important in stress responses in female rats and the possible regulation of CRH neurons in the area (106).

## Relaxin-3/RXFP3 in Mouse Hypothalamus – Species Differences

The distribution of relaxin-3 neurons and projections, as well as the distribution of RXFP3 mRNA and binding sites in the mouse brain, is regionally similar to that in the rat both generally throughout the forebrain and within the hypothalamus (33, 34). Furthermore, the distribution of Rxfp3 mRNA in the C57BL6/J mouse detailed in the Allen Brain Institute Brain Atlas (see text footnote 1) using a digoxigenin-labeled RNA probe is similar to that observed in our studies using radioactively labeled oligonucleotides (34) (Table 1). However, a more detailed analysis is required to determine whether identical populations of neurons are targeted within key hypothalamus areas, such as the arcuate, periventricular, PVN, and SON of rat and mouse to assess whether relaxin-3 signaling might regulate similar neuroendocrine processes in these species.

We have conducted several studies to assess the possible role of relaxin-3/RXFP3 in food intake in mice (121). After the administration of similar or higher levels of relaxin-3 or various RXFP3-selective agonists (R3/I5, RXFP3-A2) used in studies on rats [see (76) for review], we did not observe an increase in food intake of satiated C57BL/6J mice. In more recent studies, we have observed that the icv administration of the RXFP3 antagonist, R3(B1–22)R (73) produced a reduction in the robust feeding that occurred in mice offered access to regular chow after a 4 h period of isolated housing in a novel cage without bedding and in the absence of food (Smith and Gundlach, unpublished data). These particular conditions are presumed to induce a level of stress, hypothermia, and energy deficiency in the mice, which motivates their feeding; and suggests that relaxin-3/RXFP3 signaling in mice may not influence feeding significantly under basal conditions, but may play a role under altered stress conditions. These possibilities are currently being explored experimentally.

Consistent with these pharmacological studies, however, there is no genotypic difference between relaxin-3 KO and wild type littermates in bodyweight, total food consumption, or circadian rhythm of food consumption (120, 122). These findings are in contrast to data obtained by Sutton et al. (123) in a separately derived colony of relaxin-3 KO mice on a mixed C57BL6/J/SV129 background, which displayed a markedly reduced body weight relative to wild type mice when both genotypes were fed a diet with a moderately elevated fat content. However, subsequent studies of a null mutation Rxfp3 KO mouse strain revealed no body weight-related phenotype, but did reveal an identical circadian hypoactivity phenotype to the relaxin-3 KO mouse (120, 124, 125). This suggests the relaxin-3 KO phenotype reported by Sutton et al. (123) was associated with genetic differences independent of relaxin-3/RXFP3 [see (39)].

With regard to the specificity of pharmacological studies of RXFP3 signaling in the mouse, while the presence of insulin-like peptide 5 (INSL5) and RXFP4 in the mouse brain has been reported (126), these findings have not been independently validated; and in a separate study, the presence of INSL5-sensitive receptor binding sites could not be identified (127). Furthermore, the ligand/receptor expression profile suggests the INSL5/RXFP4 system acts primarily within the gastrointestinal tract and large intestine (28, 128). While it is possible that peripheral INSL5 signaling may alter central (hypothalamic) function, at this stage no such data is available.

## Conclusion and Perspectives

A decade of research has revealed the basic structural framework of central relaxin-3/RXFP3 networks and their likely functional importance in mammalian brain (27, 39, 76); and several studies have highlighted the interaction between hypothalamic homeostatic systems and relaxin-3/RXFP3 signaling, predominantly in pharmacological and regulatory studies in the rat. Together, these studies point to a role for relaxin-3/RXFP3 in regulating hypothalamic activity, with evidence suggesting it does so via interactions with oxytocin, AVP, and/or CRH, to modulate neuroendocrine function associated with stress, feeding, and metabolism, and motivation and reward. Further studies are now required to clarify the nature of these various effects and how they are coordinated and regulated by hormonal and transmitter inputs onto relaxin-3 neurons and integrated signaling at the level of hypothalamic loops (e.g., HPA, HPG axes). Recent research strongly suggests the relaxin-3/RXFP3 system is an important regulator of the neuroendocrine axis, with potential as a novel therapeutic target for the treatment of neuroendocrine disorders and associated behavioral dysfunction.

## Conflict of Interest Statement

The authors declare that the research was conducted in the absence of any commercial or financial relationships that could be construed as a potential conflict of interest.

## Footnotes

^1^www.brain-map.org
